# Sewing shirts with injured fingers and tears: exploring the experience of female garment workers health problems in Bangladesh

**DOI:** 10.1186/s12914-019-0188-4

**Published:** 2019-01-21

**Authors:** Sadika Akhter, Shannon Rutherford, Cordia Chu

**Affiliations:** 1 School of Science and Environment, Griffith University, 170 Kessels Road, Nathan, Brisbane, Queensland 4111 Australia; 2 International Centre for Diarrhoeal Disease Research, Dhaka, Bangladesh; 3 School of Medicine, Griffith University, Gold Coast, Australia

**Keywords:** Bangladesh, RMG, Qualitative research, Female workers, Health and safety, Gender, Work environment

## Abstract

**Background:**

The ready-made garment industry in Bangladesh not only contributes to the nation’s economic development, but has created income opportunities for women, benefiting their whole family. However, these benefits come at considerable cost to the women. This research examines how the work environment and gendered family role in this conservative society affect the health of the female industrial workers.

**Methods:**

A qualitative study employed in-depth interviews (n-20) and focus group discussions with female garment workers (n-4) in two cities of Dhaka district. Further, key informant interviews (*n* = 4) with factory doctors, along with eight workplace observations were conducted to explore the lived experience of female workers’ health issues. Interview transcripts were coded in Atlas-ti, 5.2. The data were analysed using thematic analysis approach. The themes are illustrated with case narratives.

**Results:**

The female workers reported that their work has led to back and joint pain, continuous headache, eye pain and difficulty in breathing associated with inhaling fabric dust. Inadequate lighting, constantly sitting in one position without back rest and continuous noise from hundreds of machines makes them feel permanently tired. Further, the female workers reported that working in the factory and meeting the expectations of the families at home has doubled their workload. The doctors indicated that the physical work environment, their low job status and the nature of the job affect the health of female workers.

**Conclusion:**

This study found that female workers in the ready-made garment industry face a high risk of health problems. Both government and non-government organizations need to be better involved in designing interventions targeting these women, to protect them from such health risks. In addition, recognition by the whole society of the important role the women play in the economy is needed, so that support by both family and society can be improved.

**Electronic supplementary material:**

The online version of this article (10.1186/s12914-019-0188-4) contains supplementary material, which is available to authorized users.

## Background

In the 1980s, the global adoption of the open market economic policy increased industrialization in developing countries [1]. The expansion of trade through industrialization created job opportunities for women to participate in paid work especially in manufacturing industries [2]. A noticeable change in women’s participation in industrial work, which increased from 25 to 44% of the workforce, was seen in South Asia from 1970 to 90 [3]. Foreign investors employed unskilled female workers with a lower level of education to work in the garment industry in many developing countries because they could pay low wages [4, 5].

In 1971, Bangladesh became an independent country and within three decades, the ready-made garment (RMG) industry had become the second largest industry in the world [6]. The Multi- Fibre Arrangement (MFA) that imposed quotas for textiles and ready-made garment exports from developing countries to developed countries helped to grow the RMG sector in Bangladesh [7]. The RMG industry of Bangladesh has been identified as one of the top sources of ready-made garments in Southeast Asia and the volume of the export of this industry is expected to triple by 2020 [8]. It earned USD24.5 billion in the fiscal year 2014–15, which accounted for over 80% of the nation’s export earning [9].

This industry employs around 4 million workers and most of them are women with low or no educational background largely from rural areas who earn some of the lowest wages in the world [10]. Despite phenomenal growth, this industry’s reputation has been questioned because of two major industrial incidents. In 2012, a fire at Tazreen Fashions, a ready-made garment factory in Dhaka, caused 112 deaths [11] and a few months later, the Rana Plaza building collapsed in 2013, causing the death of 1129 mainly female garment workers and injuring thousands more [12–14].

Women’s participation in work has significantly contributed to reducing poverty and addressing the issue of gender equity, mostly in developing countries [15]. Research in Bangladesh has found that through employment opportunity, for example in the RMG, women have become more empowered in Bangladesh [16–18]. Moreover, for families, the income of the female garment workers increases their family’s economic position and household consumption of food, and helps them invest more in the education of their children [7, 19, 20].

However, the women who participate in unskilled work are at risk of adverse effects on their health and well-being due to the work environment and demands associated with this changing gender role [21]. Studies have found that participation in paid work increases women’s workload, family conflicts, and their vulnerability to male marital violence [22, 23]. Many studies about the effects of physical health problems of the industrial workers focuses on musculoskeletal injuries, pain, and fatigue [24–27]. Some studies have found neck, back and shoulder pain among female garment workers [28–30]. Other studies in India, Cambodia, Viet Nam and Sri Lanka report more generally that a poor working environment affects the health of female workers [31–35].

Over the last decade or so, some Bangladeshi researchers have started to explore the health of female industrial workers. Some studies on female garment workers’ have identified the types of their health problems [36–39]. Other researchers have concentrated mainly on labour rights and standards, fair labour practices, and working conditions [40–43].

Although musculoskeletal disorders (MSDs) have received the main attention in research in the field of industrial workers health, the issue of mental health at work is less explored, especially in developing countries [44]. Stress in the work environment can have a significant impact on employees health, making this issue a major public health problem [45]. According to one study garment workers suffer from health problems, such as work stress and anxiety in the RMG setting in Bangladesh due to the physically demanding work, time pressure and verbal abuse [46].

Though this type of evidence is needed in Bangladesh, an insightful ethnographic narrative of the work environment and its impact on the health of the female workers is absent from the research because the research approaches have mainly been limited to quantitative methods. Thus they are unable to provide information on how workers identify and experience their health problems from participating at work.

The work and health of the factory workers need to be studied beyond the closed system of the factory floor. More emphasis needs to be given to the wider interactions of workers’ everyday life and health [47]. This paper explores through a qualitative lens how female workers see their experiences of work and health problems in the RMG industry in Bangladesh in relation to the working environment. It further attempts to complement the workers’ understanding of their health problems with the perspective of the factory doctors. The objectives of the study are: 1) to understand how female workers perceive how the work environment and production quota system affect their health 2) to explore female workers’ perspectives about their health, income and family life both in the past as well as the present.

## Methods

### Theoretical approach

The underpinning theory of this research is health geography to explore how working conditions and gender play an important role to determine the health of the women [5, 35, 48–51]. This research explores how and why the environment at work and gender roles affect the health of female workers. One of the key elements of health geography is that there are multiple determinants of health which include biological factors, human behaviour and the environment [51–53]. We attempt to understand female workers health problems throughout their life experiences, which include their work at the workplace and at home.

### Study site, design and participant recruitment

The research was conducted in two cities of Dhaka district in Bangladesh in four export oriented ready-made garment factories with 12,000 workers, 80% of whom are female. Participants of this research were recruited from two factories in Mirpur and two from Savar. The rationale for selecting two cities to collect data was to identify cross-site issues and major patterns of data to enrich the research [54, 55]. Fieldwork was conducted for eight months from December 2015–July 2016.

This study took a qualitative approach in order to gain a holistic picture of the female workers’ situation in their work environment, providing an opportunity for female workers to listen to their voices relating to their lived experience of health problems at work. A total of 20 in-depth interviews (IDIs) and four FGDs (focus group discussions) [a total of 36 female garment workers participated in the FGDs] were conducted with married women of reproductive age (18–49 years) who were working in the RMG with at least one year of work experience and who resided in the selected study areas. In Bangladesh those under 18 years are considered children and as such this study recruited female workers over 18 years [56]. All of the women were Muslim and married except two who had divorced. The female workers had education up to grade five while many had no formal education. Only one of the participants completed education up to grade 10.

Eight observations and four key-informant interviews (KIIs) with doctors employed by the factory were conducted. This study attempts to understand the individual and group perspectives of how female workers experienced their health after starting work in the RMG industry [57]. Observations were done at the factory and where the female workers live and this helped to explore where and how the women work, the physical conditions and processes of the workplace such as the dining room, toilet facility and rest breaks, and the equipment they work with.

Purposive sampling was used to identify and select individuals or groups who have knowledge and experience about the research topic and are able to provide rich-information relevant to research questions [55, 58, 59]. A further criterion was whether the participants were available and willing to participate in the study [60]. The purposive sampling method supports good communication between the researcher and enables the study participants to express their opinions about the study topic in an articulate, expressive and reflective manner [61]. The researcher contacted female garment workers with the help of local NGOs and recruited them independently. All of the FGDs and IDIs were conducted at the home of the study participants while KIIs were conducted in the offices of the informants. To conduct the FGDs, IDIs, and KIIs an appointment was made to fix a date and time to the conduct the interviews.

### Data collection and analysis

IDIs and FGDs with female workers were conducted at their homes or at a convenient location recommended by the workers. The conversations were started with an introduction about the purpose of research. Data were collected with a semi-structured topic guideline (Additional file 1) to develop conversations instead of having interviews with a list of questions [62]. The topics that were initiated during interviews and FGDs with female workers were: (1) work at the factory and at home (2) health and safety problems at work (3) working environment and perceived factors that influence health problems (4) seeking treatment for health problems. However the conversations frequently went beyond these topics to many topics of concern to the women themselves. A separate guideline was developed for KIIs. Appointments were made over the phone to conduct the KIIs in a private room in their work place.

In the FGDs, female workers discussed their experience of health problems in general. The FGDs were conducted by two female researchers: one facilitated the discussion while the other assisted with the logistics of gathering the women and taking notes as needed [41]. All interviews were recorded with a digital audio recorder and each interview and FGD lasted approximately 45 to 60 min. All the KIIs, IDIs, and FGDs were conducted in Bengali by the first author who is a native speaker and translation of data was done by the first author. FGD participants were provided with a snack and drink to show appreciation for their time. IDI participants received a small gift.

Data was analysed through a qualitative thematic analysis process [63]. To begin analysis, all audio recordings were transcribed and verified. All transcripts of interviews, FGDs and field notes were entered into Atlas-ti version 5.2 before coding. The data were coded based on categories and patterns by reading and re-reading the transcripts. Two researchers independently coded three transcripts and developed a codebook for this study. The remaining interviews were coded by the first author using the codebook. The topic of the interviews helped the researchers to explore issues relevant to the analysis of how working environment and gender roles may affect the health of the female workers and coded concepts related to health, injury, type of work and physical labour.

### Ethics

Written informed consent was obtained from all study participants before conducting and tape recording the interviews. The purpose of the study, potential benefit and the right to withdraw from the study were explained to study participants before the interviews were conducted. To ensure confidentiality and anonymity, individual identification were removed.

## Results

The findings represent a description of female workers’ health status and their understating about their health problems both individually and as a group. Quotes from the study participants present the female workers’ narratives. From the four sampled factories, cases are presented based on the in-depth interviews to represent all study participants. The cases show who the factory workers are, from where and why they migrated, their health problems, income, family expectations, job options, and future plans.

### Gender division of labour, work environment and risks of health problems

The production room has both men and women working together. However, the women said that the production manager (PM), the floor-in-charge who works under the PM and the line supervisors on the production floor are all men. All the sewing machine operators and helpers are women. Operators and helpers are the two lowest positions on the production floor. The female workers reported that they cannot share their health problems with their supervisors comfortably as they are “men”. In this regard one woman said, *“When we have our periods, some of us get sick from excessive discharge of blood, we need to go the toilet to change frequently but we cannot share our problems because we are embarrassed and they are all men around us.”* Thus, they are unable to ask to adequately address their health needs.

The in-depth interviews with the female workers and the work site observations identified how the conditions of the work environment can affect their health. The female workers reported that the floor is overcrowded and they have difficulties breathing due to fabric dust. There is no air conditioning, although there are electric fans on the production floor and the workers suffer heat exhaustion during summer, as the number of fans is not sufficient. They suffer due to the smell of sweat, the noise from 500 machines in one large room, poor lighting, and having to climb up to 10 storeys of stairs twice a day, despite the presence of lifts. The lifts are reserved for the owners, managers, and their visitors.

This story describes a typical work day and its impact on the health and wellbeing of a female worker who works as a machine operator. She had finished primary education (five years), started her job at the factory as a healthy woman around four years before the interview. She explained how her work as a machine operator creates health problems. She sits on a small wooden tool without a backrest for 10 to 12 h each day. She sews the cuffs of shirts, one hundred per hour for the whole day, which puts constant pressure on her fingers and wrists. This sitting arrangement at work causes constant pain in her back. The work is monotonous and repetitive. The poor lighting, inhaling the fabric dust, and heat in the factory environment may contribute to the headaches and eye irritation. She indicates that the factory provides a face mask to protect workers from inhaling fabric dust, but she does not use it because it feels hot.

Despite there being doctors available, the workers do not have opportunity for regular medical examinations to check their health status. She added, *“I was not as lean and thin as I am now. I lost weight after starting to work at the factory and lost my beauty. When I see myself in the mirror I cannot recognize myself. I do not get enough time to sleep and rest. Now I always have dark spots under my eyes.*”

### Physical illness and Panadol

The work hours are very long. The women reported that they are officially required to work for eight hours, but the production quotas are so high that they routinely work ten to twelve hours to meet the quota. The extra hours to meet the quota should be paid according to the regulations, but not all factories participating in this study fulfil this obligation. The female workers reported that they also do not refuse to do paid overtime (above the normal hours) as they can earn some extra money. However, these extra hours of work make them feel sick. Female workers reported that they suffer from headaches, eye complaints, body aches, and fatigue. They further reported that these physical illnesses have become ‘normal’ in their lives and they can live without treatment.

*“I started working at this factory five years ago as a helper. Now, I work as a machine operator. I sew shirts.”* says one respondent. Her monthly salary is 7000 taka (US$87) and with overtime, she earns 10,000 taka (US$125). She works 2–4 h overtime each working day, six days per week (total 28 days/month) to earn the extra money. She further added *“I do overtime to earn some extra money to pay for food, house rent, and education for my children.”* This work gives her the opportunity to earn extra money but she suffers from headaches, muscular pain, and back pain. She further added, *“I have been working for last five years with these pains. Now I don’t think that they are pain, I even don’t go the doctor. As I work as a machine operator [operating with pedals], I always feel pain in my legs. I buy Panadol from the pharmacy and I take this tablet when I cannot endure the pain in my leg.”*

Another woman explained, *“Panadol becomes our main food to survive from all of our physical illness and pain. Every women who works at the factory they carry Panadol and eat it like rice because we all suffer from different pains in our body and Panadol helps to survive.”*

### “Tigers on the land and crocodiles in the water”

All of the women stated that meeting the demands of their job and taking care of their family members is a constant battle in their lives. The activities of women within the household include taking care of children and other family members, specially husbands, preparing and serving food to family members, washing and cleaning. All the female workers reported that the factory work gave them opportunity to earn money but their work load is doubled. They work at the factory but they also need to do all the household work. They further stated that they do not receive enough support and care from their husbands when they return home after work. Instead, they feel pressure to do all the household work to make their husbands happy.

### The story of a quality inspector

“*I started working at the garment factory three years ago. Now, I work as a quality inspector. I check the quality of every sewn trouser. I need to do the work constantly by standing in front of a table. I always feel pain in my feet for standing for a minimum of 10 hours per day. Now it is the holy month of Ramadan. We fast from very early in the morning to evening. We get one hour break during the evening to break our fast. We get a long vacation (10days) during our Eid [the biggest Muslim religious festival at the end of Ramadan]. We are working 7 days in this holy month of Ramadan to compensate for the days we will get as vacation during Eid. In fact we need more rest during Ramadan as we are fasting. Instead we work more this month to compensate for the days off.*” She added, “*When I return home after work I need to do household work. My work at home as a woman remains the same despite working at the factory. Last week I fasted, I worked the whole week and after I returned home I felt so tired that I couldn’t cook. I lay down and fell asleep. When I sleep sometimes I cannot move from one side to another side. I feel that I am paralysed as I work standing up. My husband became angry with me as I couldn’t cook for him. I need to make happy every day two of my supervisors, one at work and one at home. No one wants to listen to your pain of tiredness. Both of them want us to work*.” She further lamented, “*I am working hard at the factory and at home but most of the time I cannot make happy either my supervisor or my husband.”*

### Paid work choices, longevity and future plans

All of our study participants reported that working in the garment factory is better than working as a domestic servant, a sector where unskilled woman are generally employed. They further stated that they had no choice except to work as a domestic servant or as a factory worker. Factory work is like a profession, it gives them social identity. They can do extra work to earn extra money but in domestic work they cannot earn extra money. Though they work most of the weekends in the factory, they do get some holidays.

#### The story of a helper

She came to Dhaka with her husband and children for work. She can only write and read her name. She used to work as a housemaid in Dhaka. But the employer did not behave well towards her and did not give her food. The salary was very low. She was thinking about changing her job. She talked with one of her neighbours who worked at a garment factory. Her neighbour took her to the factory to meet her supervisor. The supervisor told her to go to the factory with her national ID card and there she got a job working as a helper. She folds the finished shirts to pack, standing all day. After starting to work in the factory as a helper, she always feels pain in her waist. The doctor said she needs to take breaks during work but running to meet the production quota provides little opportunity to take a rest during working hours.

All of our study participants shared their future plans. Every female worker said that they would work ten years in the garment industry. Many of them had already spent five years and would work five more years. After this time they will return to their own village. They are trying to save some money to buy some land in their village and to build a house. Some of the women reported that they establish a tailoring business in their own village after they leave the job. All of the women said that after working ten years in the garment factory, they will not do any factory work. They want to rest and spend time with their family.

#### The story of a machine operator

She completed her education up to grade five. She says,“*We sew shirts with our tears and injure our fingers due to needle punctures. If you work at the garment factory it will give you some money but it will take your health. Every day my fingers get injured due to needle punctures. I wear a needle guard to save my finger but I can work faster if I do not put the needle guard on my finger, but that causes finger punctures. Now I do not care about needle injury. I just think I need to work hard to earn more money to save some money for the future of my family. No one can work in a factory more than ten years because you will lose your physical strength, energy and health after ten years due to the nature of hard work in this industry.”*

### The factory doctors’ perspectives

The key informant interviews focused on the health services provided by the factory clinics and the health problems facing the factory workers. One of the key informants described how the factory clinics run, *“The factory provides health services to the workers through factory clinic. The clinic has a sick room and nurses. We [Doctors] are available in the afternoon but the nurses are available the whole day. If you [pointing to the researcher] visit the factory floor you will see first aid boxes are available on each floor to treat them for small injuries, fever, and headache. We have paramedics on each floor to treat the workers. When the paramedics cannot treat the problems they refer the workers to the factory doctor for further treatment.”*

A factory doctor explained the health problems associated with the work of the factory workers, *“The female workers suffer from mainly headache, eye pain, back and joint pain and weight loss. The first three health problems occur due to the repetitive nature of the work. The weight loss is very common among the female workers. The reason for weight loss is not getting opportunity to take enough rest and not eating enough food, On the contrary they work hard to earn money to run their family. We can only advise them to take enough food and to have enough rest.”*

Another doctor identified health problems related to the job position: *“The women who work as machine operator they come to the factory clinic with a complaint of suffering from dry cough. The machine operators inhale dust of fabric for continuously sewing clothes. The machine operators also suffer from injury due to needle punctures. The factories provide needle guards and masks but they do not use them. Sewing machine operators also suffer from the back, neck, and shoulder pain. The women who work as a quality control inspector and ironers suffer mostly from musculoskeletal pain in the knees and thighs due to working for long hours standing. Some women complain about losing hearing. They do not lose hearing power but noises of machine make them feel headache.”*

The doctor further reported that the factory clinics provide limited health services. They mostly prescribe pain killers to treat the illness of the workers, along with some health advice*.* The doctors were asked about the longevity of the workers working tenure. In response to this question the doctors reported that the female workers cannot work more than ten years because they get physically exhausted and unfit to work.

## Discussion

This is one of the few qualitative explorations of female workers’ health problems from the perspective of the workers and healthcare providers using interviews, FGDs and observations with female workers and doctors in the RMG factories in Bangladesh.

The workers of this study shared their own experience of health problems after starting work in the garment factory. The female workers reported that participation in paid work made them more vulnerable to physical illness. Interviews found various types of physical illness among women workers. These physical health problems include headaches, eye pain, musculo-skeletal pains and fatigue. It further revealed that garment work is also so physically demanding that women cannot work more than ten years. These findings are consistent with other research which found that the highest proportion of female workers quit factory work before they reach 40 [37, 64, 65]. The workers reported that getting sick and injured is an everyday phenomenon in their lives. They do not even go to the factory clinic for treatment; rather they buy Panadol and treat themselves. The doctor of the factory clinics agreed about the health problems and treatment procedures reported by the female workers. Furthermore, the doctors acknowledged that the female workers cannot work at the factory more than ten years due the nature of the job and work conditions. The workers work under stress which effects their health but the factory provides very limited health services to treat the workers, and this is consistent with the findings of other studies [14, 66, 67] . The doctors also described how the different job positions create different health problems among the female workers.

Further, this study describes the issues faced by the female workers due to the gendered division of labour at the RMG factories. As the supervisors of the female workers are men, the women feel unable to discuss their health needs, including their needs during menstruation. Further, this gendered division of labour extends to their home life where their husbands expect them to fulfil their domestic obligations despite long, physically demanding hours at work.

These women do not have opportunities to work in other industries except working as a domestic servant. The women expressed their concerns that work at the garment factory is so hard and exploitative. Nevertheless, thousands of these women work in this industry because it has more social status and they can earn extra money by doing overtime. They prefer to work in the RMG sector rather than as a domestic servant. Previous studies have also identified similar findings [35, 68].

The findings indicate that female workers typically migrated from rural areas to work in the garment industry to meet their financial needs. This finding is consistent with other research which found that poverty was the main driving force for women to participate in paid work in a factory [69–71]. Employment in the RMG has brought economic changes in the lives of the women despite the physically demanding nature of the work. However, based on the narratives of the female workers, this research found that the economic benefits come at the cost of the health of the female workers. These women are an important labour force for the country but RMG employment has the potential to make women sick and in the long term lead to a health burden for the country. The government needs to make policies and enforce regulations that protect and promote health and safety for female industrial workers. The RMG sector heavily relies on unskilled and uneducated village women. To maintain the future economic growth and sustainability of the industry, it is important to keep female workers productive by taking care of their health. This is particularly urgent in a context in which women state that they couldn’t work more than five to ten years in this sector due to their physical illness, which was confirmed by the factory doctors. In addition, the dual burden of work and household responsibility means that there needs to be more support for the female workers, so that they can balance their household work with their factory work.

Moving forward, the following steps are recommended based on the findings of this study. Firstly, since globalisation brings economic benefits to the whole nation as well as to these women but at the cost of health problems expressed by the female workers, the government, international trade community, and development organizations all need to cooperate to regulate the labour market to create a healthy and safe working environment for this important labour force.

Secondly, the country needs to build capacity to monitor the working environment and related health problems of the workers. The government needs to work with factory owners, international organizations, trade agencies and donors to develop a good monitoring system to analyse and address the key factors of occupational health problems in order to address the health and safety issues identified. There are examples from history in other settings of how changes in processes (such as job rotation) can enhance worker health and productivity.

Thirdly, these women need family support; especially families who need to listen to the voice of the women about their problems. Societal interventions should be designed to address issues of gender roles at home to reduce the double burden of work.

Finally, although Bangladesh is emerging as a low-middle income country, rural poverty is still a major problem and rural women lack opportunity for education, lack independence and lack access to property [21, 72, 73]. More interventions for women’s social development are needed to build skill and capacity for women so that they can advance to more rewarding positions in industrial enterprises. All of these policy changes will also require the participation of women workers in dialogue with policy makers for policy formulation.

### Limitations and strengths

This study has some weaknesses and strengths. Recruiting female study participants was sometimes challenging due to their time constraints. Most of the interviews were conducted at night and weekends when the workers were tired or distracted. Further, a small gift was given to the IDI participants and snacks were served to the FGD participants as to acknowledge their time for participating in this study. There was minimal opportunity that the gift or snacks could affect the findings as the respondents were not informed about the small gift will be given or snack will be served when they were recruited for this study. The gift was given at the end of the interview and snacks were served also at end of the FGDs. Thank you so much for this suggestion.

All the interviews were conducted in local language (Bengali), transcripts were prepared in Bengali and findings were translated to English for analysis. Some concepts may not be easily translatable.

This study was conducted in four factories in two cities of Dhaka district in Bangladesh; the results may not be transferable to other settings in Bangladesh. However the workers’ narratives of their own experience suggest avenues for further exploration.

The finding of this study is limited to workers who are currently working with health problems; health issues of the workers who quit weren’t the scope of the study, thus did not sample those workers. There is limited published research on female workers health problems in RMG in Bangladesh through qualitative research. Consequently this study makes an important contribution to the international academic literature.

## Conclusion

The stories of the RMG female workers’ health problems are powerful because they reveal the struggle for economic mobility in an environment which is competitive and detrimental to their long-term physical health, well-being, and a threat to the sustainability of their employment.

The working conditions act to gradually destroy the workers’ health. Few of the women are able to continue the work beyond five to ten years because of the health problems they experience. The competitive nature of the industry and the weak support for workers from government and industrial leaders means that the labour codes for health and safety are normally ignored. Further, in this conservative society, women bear heavy expectations from their family – husband, children, in-laws – in terms of housekeeping and food preparation. These expectations lead them to neglect their own health in order to look after their family as tradition expects.

What is needed is for government in particular, and society in general, to recognise the important role these women play in the national economy and provide support for them, both in terms of their safety and health in the workplace and in terms of the broader societal expectations placed upon these workers who put in very long hours.

## Additional file

Additional file 1: Guideline of IDI/FGD. Guideline of an In-Depth Interview/FGD with Female Worker. (DOCX 17 kb)

## Abbreviations

FGD: Focus Group Discussion
IDI: In-depth Interview
KII: Key Informant Interview
MFA: Multi- fibre Arrangement
RMG: Ready-made Garment Industry
