# Supplementary material for: Sewing shirts with injured fingers and tears: exploring the experience of female garment workers health problems in Bangladesh
Source: BMC Int Health Hum Rights. 2019 Jan 21;19:2. doi: 10.1186/s12914-019-0188-4 (PMC6341570; doi:10.1186/s12914-019-0188-4)
Supplement: Supplementary file 1 — Guideline of IDI/FGD. Guideline of an In-Depth Interview/FGD with Female Worker. (DOCX 17 kb) [file 12914_2019_188_MOESM1_ESM.docx]

**Socio- demographic profile**

Date: ____________________

Interviewer Name: _____________________

Respondent name: ______________________________

District: ______________

Upazila: ______________

Village: _________

Age:

Occupation:

Religion:

Education:

Marital Status:

Husband’s Age:

Husband’s Occupation:

Mother is living in maternal (natal) home, in-law’s home, or independent home

Household

Total number of household members living in the same house : __________________

Number and ages of children:

Economic indicators:

Electricity: Yes No

*House characteristics*

- Composition of floor:

- Composition of roof:

- Composition of walls:

Is there child still breast feeding_____________ Yes No

Information Related to job, usual work and earning:

**Information related to work: purpose and rationale**

*Asking about their job and work is a good way to engage the respondent and begin the process of building rapport because this is a topic about which each one of them has something to say, so it will put them into a position which will help the flow of the conversation.*

i) To establish rapport with respondents through questions those are relatively easy for them to answer

ii) To know respondents’ current position, content of work and their earning as a basis for starting the dialogue on job and everyday work at the factory within the larger sphere of concern of this study, (which we call health and safety issues’)

iii) It will also help to get a picture of the typical day of garment worker’ s life at the workplace and at home

I could start the discussion by saying, ‘I’d like to start by asking when you joined in your current factory to work?’

Then ask why did you decide to work in a garment factory?

How did you get this job?

Did you receive any appointment letter when you joined in this factory?

What is your current work position or role?

When do you go to work every day in the morning?

What type of work do you do at the factory usually?

How long do you work at the factory? (Probe: hours, overtime)

When do you get a break during work? How many breaks do you usually get in a day?

What do you eat during your breaks?

**Information related to transport from home to work and work to home:**

*In this section I will expand the focus from job and work to the area of transportation and safety issues. It will set the stage for the next two topics which are aimed at understanding health and safety issues to get the larger picture of the study topic. They are open-ended questions with guided probing.*

Introducing the topic: ‘Now I would like to ask you about things related to your work and transport. Can you tell me when do you return home after work and how do you return home after work?’

If she says, she goes to the factory walking, then I will ask ‘Tell us in detail your experience of walking in the street’ (Probe: teasing, comments and her feeling and coping strategy about it)

How do you manage transport to return home at night?

What are the safety problems you face at night in returning home?

How do you manage the transport cost if you return home at night?

What transport facilities do you have from the factory?

**Information related to health problems:**

*First I will introduce the topic to the respondent by saying: ‘Now I would like to ask you about health-related problems. We will discuss health-related problems in detail, care-seeking practices and economic constraints on treatment action choices’.*

I will start the discussion by saying: ‘Many women have many health-related problems in their life; I would like to know about your health-related problems’.

Can you tell me what are the health problems did you have before working in the garment factory?

What health problems do you have now after joining the factory?

Where do you go for treatment when you have any health-related problems?

When was the last time you got sick due to your work? What was the problem?

Where did you go for treatment? How much did you pay for your treatment?

How did you manage the money to bear the treatment cost?

How do you manage your time when have your menstruation?

How many toilets do you have at your factory?

What is the condition of the toilets at your factory?

What are support did you receive from your supervisor?

What support did you receive from your employer?

How do you get support from your employer when you have any health/work related problems due to the work? (Probe: behaviour of the supervisor, support to get leave, emotional/mental support)

**Information related to safety issues at work and home:**

*Introduce the topic: I would like to talk to you about safety issues you encounter at your workplace. I will start by saying ‘Now I would like to learn about how the respondents’ view of the problems of safety at her workplace’.*

What are safety problems do you have at home?

What do you do to solve the problem of your safety?

**Information related to household work at home:**

*Introduce the topic. We discussed your job, transport, health and safety problems, now I would like to talk to you about your family and household work. I will start by saying ‘Now I would like to learn about how you manage your work at home beside your work at the factory’.*

Can you tell me who works in the household when you work at the factory until midnight?

Who looks after your children when you are at the factory?

What type of work do you need to do at home after you return from work?

How does your husband support you at home after return from work?

What safety problems do you have at home?

What challenges do you have as women in managing your work at the factory and at home?

**Information related to suggestion about health and safety issues:**

What needs to be done to improve your working condition at the factory?

Do you have any training related to health and safety issues?

What type training do you need about health and safety issues?
